# Whole transcriptome RNA-seq reveals key regulatory factors involved in type 2 diabetes pathology in peripheral fat of Asian Indians

**DOI:** 10.1038/s41598-021-90148-z

**Published:** 2021-05-20

**Authors:** Aditya Saxena, Nitish Mathur, Pradeep Tiwari, Sandeep Kumar Mathur

**Affiliations:** 1 Department of Computer Engineering and Applications, Institute of Engineering and Technology, GLA University, Mathura, 281406 India; 2 Department of Endocrinology, Sawai Man Singh Medical College and Hospital, Jaipur, 302004 India; 3 Department of Chemistry, School of Basic Sciences, Manipal University Jaipur, Jaipur, 303007 India

**Keywords:** Computational biology and bioinformatics, Drug discovery, Genetics, Systems biology, Diseases, Endocrinology, Health care

## Abstract

The prevalence of Type 2 Diabetes has reached an epidemic proportion particularly in south Asian countries. We have earlier shown that the anatomical fat distribution, termed ‘thin fat phenotype’ in this population indeed plays a major role for their T2D-predisposition it is indeed the sick fat or adiposopathy, which is the root cause of metabolic syndrome and diabetes and affects both—peripheral, as well as visceral adipose tissue compartments. In present study, we have attempted to unravel the altered regulatory mechanisms at the level of transcription factors, and miRNAs those may likely accounts to T2D pathophysiology in femoral subcutaneous adipose tissue. We prioritized transcription factors and protein kinases as likely upstream regulators of obtained differentially expressed genes in this RNA-seq study. An inferred network of these upstream regulators was then derived and the role of TFs and miRNAs in T2D pathophysiology was explored. In conclusions, this RNS-Seq study finds that peripheral subcutaneous adipose tissue among Asian Indians show pathology characterized by altered lipid, glucose and protein metabolism, adipogenesis defect and inflammation. A network of regulatory transcription factors, protein kinases and microRNAs have been imputed which converge on the process of adipogenesis. As the majority of these genes also showed altered expression in diabetics and some of them are also circulatory, therefore they deserve further investigation for potential clinical diagnostic and therapeutic applications.

## Introduction

Type 2 Diabetes (T2D) is a chronic, complex disorder which is caused by defective insulin secretion by pancreatic β-cells as well as weak insulin action to its responsive tissues. Worldwide 462 million individuals are suffering from T2D which corresponds to 6.28% of the world’s population^1^. Southeast Asian countries, such as China (88.5 million individuals with type 2 diabetes), India (65.9 million), Indonesia, Malaysia, Thailand, and Vietnam are also experiencing an upward trend in its prevalence^1^. Pathophysiologically T2D is characterized by hyperglycemia due to disturbance in glucose and fat metabolism; besides, the role of incorrect fat storage in adipose depots is also being realized a noteworthy determinant in the overall aetiology. Indeed, the predisposition of south Asians for T2D has been ascribed to their typical ‘thin fat phenotypes’—the accumulation of higher visceral fat (central obesity) due to relatively small peripheral fat compartment^2^. The reason for these anatomical features is likely to be genetic as even newborns in these populations show such fat distribution^3^.

Recently we have conducted a gene expression DNA microarray study of femoral subcutaneous adipose tissue in Asian Indian diabetics and non-diabetics and could foresee that peripheral fat undergoes a similar pathophysiological alteration; those have also been observed in visceral fat of T2D individuals^4^. It is however in contrast with the classical belief that the peripheral fat is protective in nature^5^. We used Weighted Gene Correlation Network Analysis (WGCNA) and found several modules of co-expressed genes showing significant correlation with intermediate traits of T2D and metabolic syndrome such as TNFα, hsCRP, Serum Creatinine, IL6, NEFA, VLDL, LDL, HDL, blood glucose, Hb1Ac, adipocyte size, insulin, HOMA-B, HOMA-IR, leptin, adiponectin etc. These results strengthen the belief that it is indeed the sick fat or adiposopathy, which is the root cause of metabolic syndrome and diabetes and affects both—peripheral, as well as visceral adipose tissue compartments^6^.

We further attempted to link the molecular basis of aforementioned thin fat phenotype in Asian Indian diabetics with that of a monogenetic disorder, lipodystrophy which shows marked similarity with T2D in clinical presentation^7^. A significant overlaps between physical and functional protein-protein interaction networks of differentially expressed genes (DEGs) in peripheral fat of diabetics and annotated lipodystrophy genes was found. To some extent, we could be able to stratify patients in terms of differentially expressed lipodytrophy genes and henceforth opined that functionally disturbed expression of lipodystrophy genes might play a role in T2D pathogenesis.

Transcription factors (TFs) are proteins that bind to the DNA and help initiate a program of increased or decreased gene transcription. Many human diseases have been associated with mutations in transcription factors for example mutation in hepatocyte nuclear factors (HNFs) results in a rare form of diabetes called MODY (Maturity onset diabetes of the young)^8^. As therapeutic modulation of TFs by drugs may lead to dramatic changes in overall gene expression pattern and could ameliorate the disease condition, identification of key TFs in T2D pathophysiology therefore promise toward the development of better anti-diabetic agents.

microRNAs are short noncoding RNAs which degrade those mRNAs whose product are not required in normal cellular physiology. Conversely, an altered miRNA expression pattern leads to a derangement in cellular physiology. Various human microRNA–disease associations have been validated experimentally and this knowledge can help us in the functional interpretation of RNA-seq datasets.

In present study, we have attempted to unravel the altered regulatory mechanisms at the level of transcription factors, and miRNAs those may likely accounts to T2D pathophysiology in femoral subcutaneous adipose tissue. We conducted whole transcriptome RNA-seq on a subset of non-diabetic, and diabetic peripheral subcutaneous adipose tissue samples which we have earlier used in our gene expression microarray study^6^. We have also prioritized transcription factors and protein kinases as likely upstream regulators of obtained differentially expressed genes in RNA-seq study. An inferred network of these upstream regulators was derived and the role of TFs and miRNAs in T2D pathophysiology was explored in the light of existing literature.

Protein kinases (PKs) have become the second most important group of drug targets after G-protein coupled receptors as deregulation in majority of signal transduction pathways have been found to be associated with abnormal phophorylation by kinases^9^. We therefore attempted to derive a minimal molecular pathway from the inferred network, comprising top-ranked TFs, PKs, lipodystrophy genes, and the central mediator of adipogenesis—PPARγ. Three kinases were identified using network analysis and proposed as potential drug targets.

## Results

**Figure 1:**
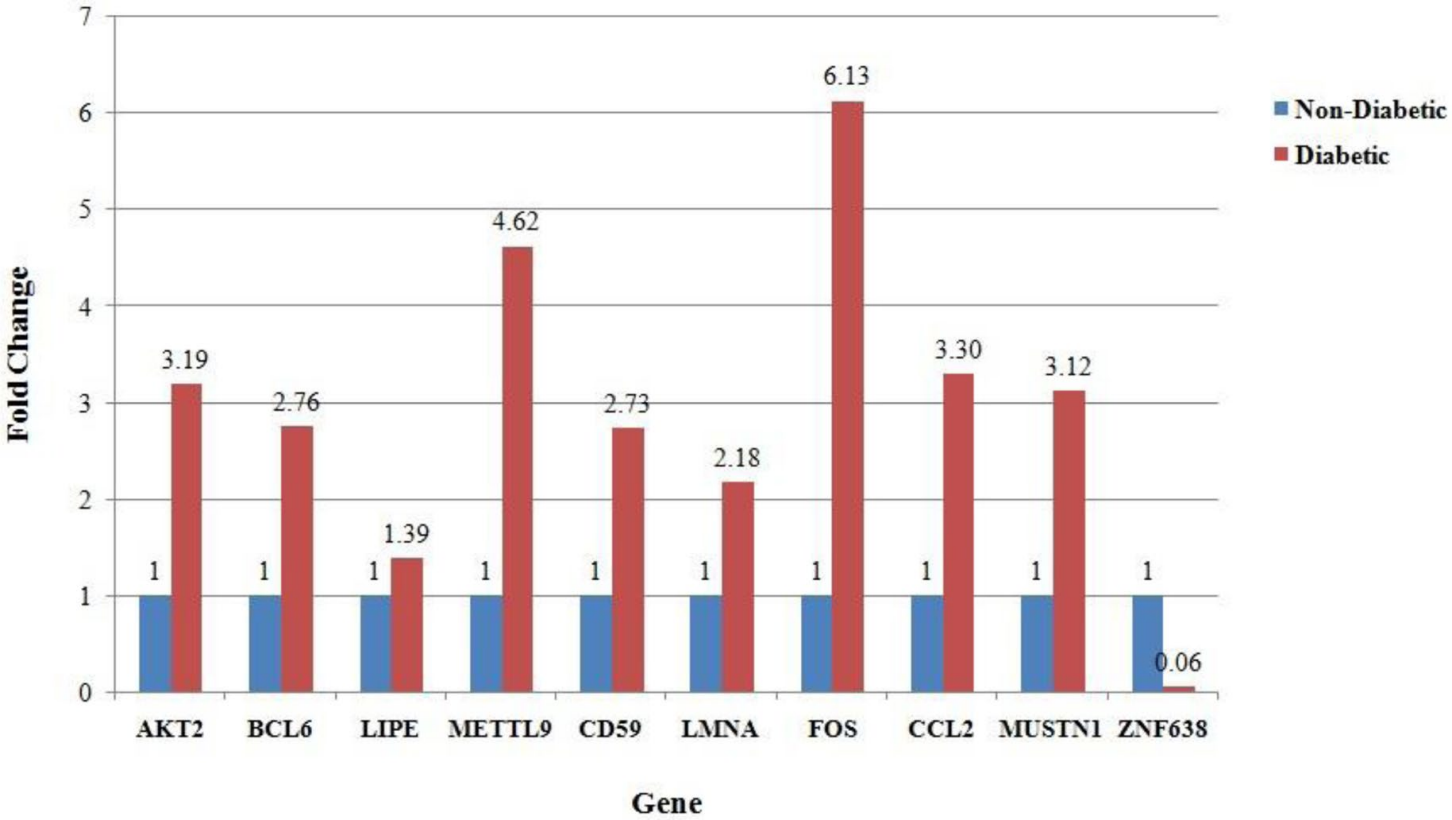
Bar plot showing fold change of selected genes in femoral subcutaneous adipose tissue in diabetic as compared to non-diabetics.

A total of 2752 differentially expressed (DE) genes were obtained by DESeq2^10^ (*P* < 0.01; logFC ± 2) between the femoral fat of non-diabetics and diabetics (See Supplementary File S1). Figure 1 shows qPCR results for the ten selected genes that validated the directionality of gene expression obtained from RNA-seq (See Supplementary File S2).

Top 2000 genes were then selected on the basis of their p-value for subsequent pathway enrichment analysis against the human collection of KEGG pathways^11^ using STRING db^12^ as it supports only a network less than 2000 nodes. We found pathways linked with glucose- and fat- metabolism (shown in bold text in Table 1) which were in line with our earlier enrichment results for microarray datasets of femoral fat.

**Table 1:** Enriched KEGG Pathways for top 2000 Differentially Expressed Genes (Log FC ±2; *P* < 0.01).

S. no.	Term description	Observed gene count	Background gene count	Strength	False discovery rate
1	**Metabolic pathways**	**259**	**1250**	**0.31**	**1.08E−21**
2	Complement and coagulation cascades	46	78	0.76	3.81E−15
3	**Cholesterol metabolism**	**25**	**48**	**0.71**	**3.26E−07**
4	**Glycolysis / Gluconeogenesis**	**28**	**68**	**0.61**	**1.65E−06**
5	Staphylococcus aureus infection	24	51	0.67	1.83E−06
6	Systemic lupus erythematosus	32	94	0.53	4.27E−06
7	Biosynthesis of amino acids	27	72	0.57	7.73E−06
8	Chemical carcinogenesis	27	76	0.54	1.62E−05
9	**PPAR signaling pathway**	**26**	**72**	**0.55**	**1.82E−05**
10	Metabolism of xenobiotics by cytochrome P450	25	70	0.55	3.17E−05
11	Drug metabolism - cytochrome P450	24	66	0.55	3.64E−05
12	Retinol metabolism	23	62	0.56	4.01E−05
13	Carbon metabolism	33	116	0.45	4.01E−05
14	Steroid hormone biosynthesis	22	58	0.57	4.47E−05
15	Tryptophan metabolism	18	40	0.65	5.28E−05
16	Glycine, serine and threonine metabolism	17	39	0.63	0.00013
17	Fat digestion and absorption	17	39	0.63	0.00013
18	**Maturity onset diabetes of the young**	**13**	**26**	**0.69**	**0.00041**
19	Nicotinate and nicotinamide metabolism	14	30	0.66	0.00041
20	**Fatty acid degradation**	**17**	**44**	**0.58**	**0.00041**

We also found a total of 65 miRNAs to be differentially expressed between non-diabetic and diabetic groups (*P* < 0.05). Eight of these miRNAs have been found to be associated with type 2 diabetes, diabetic retinopathy, nephropathy, obesity, and inflammation (Table 2).

**Table 2.** Differentially expressed miRNAs associated with T2D and related disorders.

S. no.	MicroRNA	LogFC	*P* value	FDR	Diseases
1	hsa-mir-133B	1.598985	0.01495	0.043141	T2D, Diabetic Retinopathy, Inflammation
2	hsa-mir-210	2.896913	0.03054	0.079559	T2D, Obesity, Inflammation
3	hsa-mir-22	1.447518	0.040103	0.099062	T2D, Inflammation
4	hsa-mir-25	-1.70577	0.012915	0.037956	Diabetic Nephropathy, Inflammation
5	hsa-mir-27A	2.909732	0.003672	0.012565	T2D, Obesity, Diabetic Retinopathy, Nephropathy, Inflammation
6	hsa-mir-27B	-1.56703	9.97E−05	0.000488	T2D, Obesity, Diabetic Retinopathy, Nephropathy, Inflammation
7	hsa-mir-30E	-3.9385	0.000397	0.001722	T2D, Obesity, Nephropathy
8	hsa-mir-503	-2.4246	0.046336	0.110995	T2D

**Figure 2:**
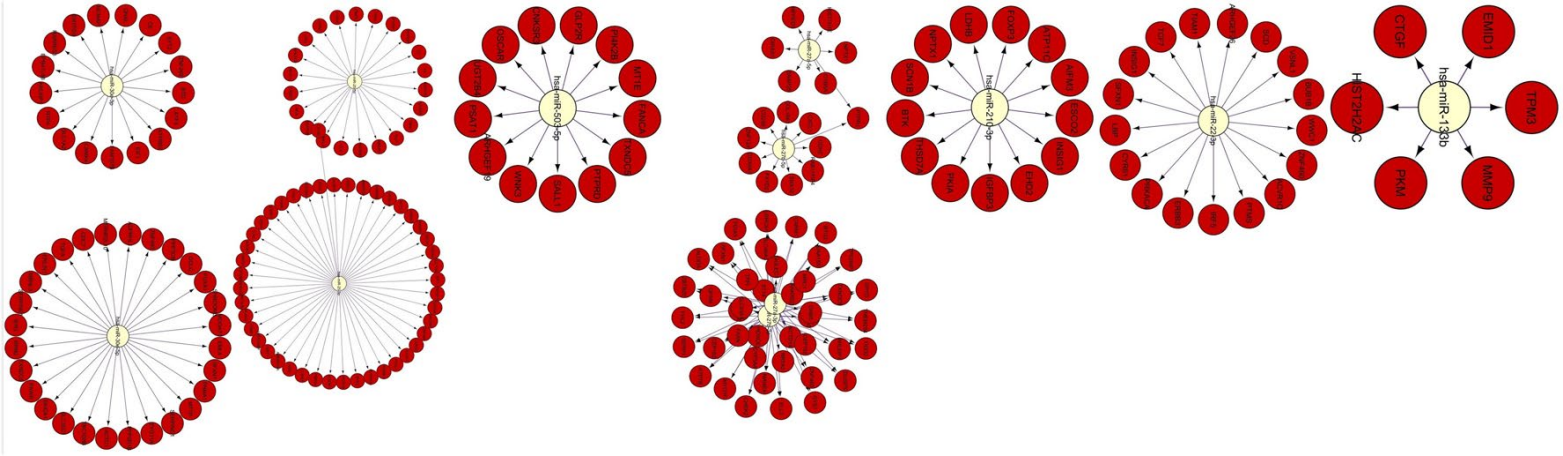
Differentially expressed miRNAs along with their differentially expressed regulated genes.

CyTargetLinker^13^ analysis further revealed that these miRNAs were regulating expression of 214 differentially expressed genes (Fig. 2).

mir133b has been reported to attenuate *GLUT4*-mediated glucose update by regulating expression of its regulator Kruppel-like transcription factor 15 (*KLF15*)^14^. mir210 also attenuates insulin signalling by targeting *PTPN1* and it’s up-regulation has been reported in T1D patients^15^. miR-22-3p activates gluconeogenesis by down-regulating the expression of transcription factor 7 (*TCF7*) which is a negative regulator of enzymes of the gluconeogenic pathway^16^. Down-regulation of miR-25 has also been observed across multiple studies in diabetic nephropathy^17^. Up-regulation of miR-27a has been reported in rat model of T2D^18^ as well as a its high circulating level has been reported in patients with metabolic syndrome and T2DM^19^. miR-27a has been observed a negative regulator of peroxisome proliferator-activated receptor-γ (*PPAR-γ*) and CCAAT/enhancer-binding protein-α, which are extremely important regulators of adipogenesis^20^. Clinically its expression was found to be positively correlated with fasting glucose level^19^. The involvement of miR-27b in diabetes nephropathy has been reported. It targets pathways related to ER stress and chronic ER stress has previously been identified in peripheral blood mononuclear cells (PBMCs) from T2DM patients^21^. Lin et al.^20^ has found a reduced expression of miR-27a and –b during adipogenic differentiation of 3T3-L1 cells in a microarray study, which was correlated with an increase of expression of *PPARγ*. miRNA-27b also contributes to lipopolysaccharide-mediated *PPARγ* mRNA destabilisation^22^. miR-30 has also been found down-regulated in patients with type 2 diabetes^23^. Deregulation of miRNA-503 has also been reported to contribute to T2D–induced impairment of endothelial function^24^. It also shows differential expression between T2D from obese-T2D patients^25^. All the diabetes-specific miRNAs were circulatory and therefore could be further followed up for their potential use as biomarkers. We also found differential expression of other miRNAs such as hsa-mir-6784, hsa-mir-4707, hsa-mir-647, hsa-mir-3652, hsa-mir-6757, hsa-mir-1-1, hsa-mir-3671, hsa-mir-6505, hsa-mir-1229, and hsa-mir-LeT7F1. The deregulated expression of hsa-mir-1-1 has been found in myocardial infarction and other cardiac complications. Similarly, hsa-mir-647 was also associated with ischemic stroke. The expression of hsa-mir-1229 was linked with Colorectal Carcinoma. The down regulation of hsa-mir-LeT7F1 has been reported in many cancers.

**Figure 3:**
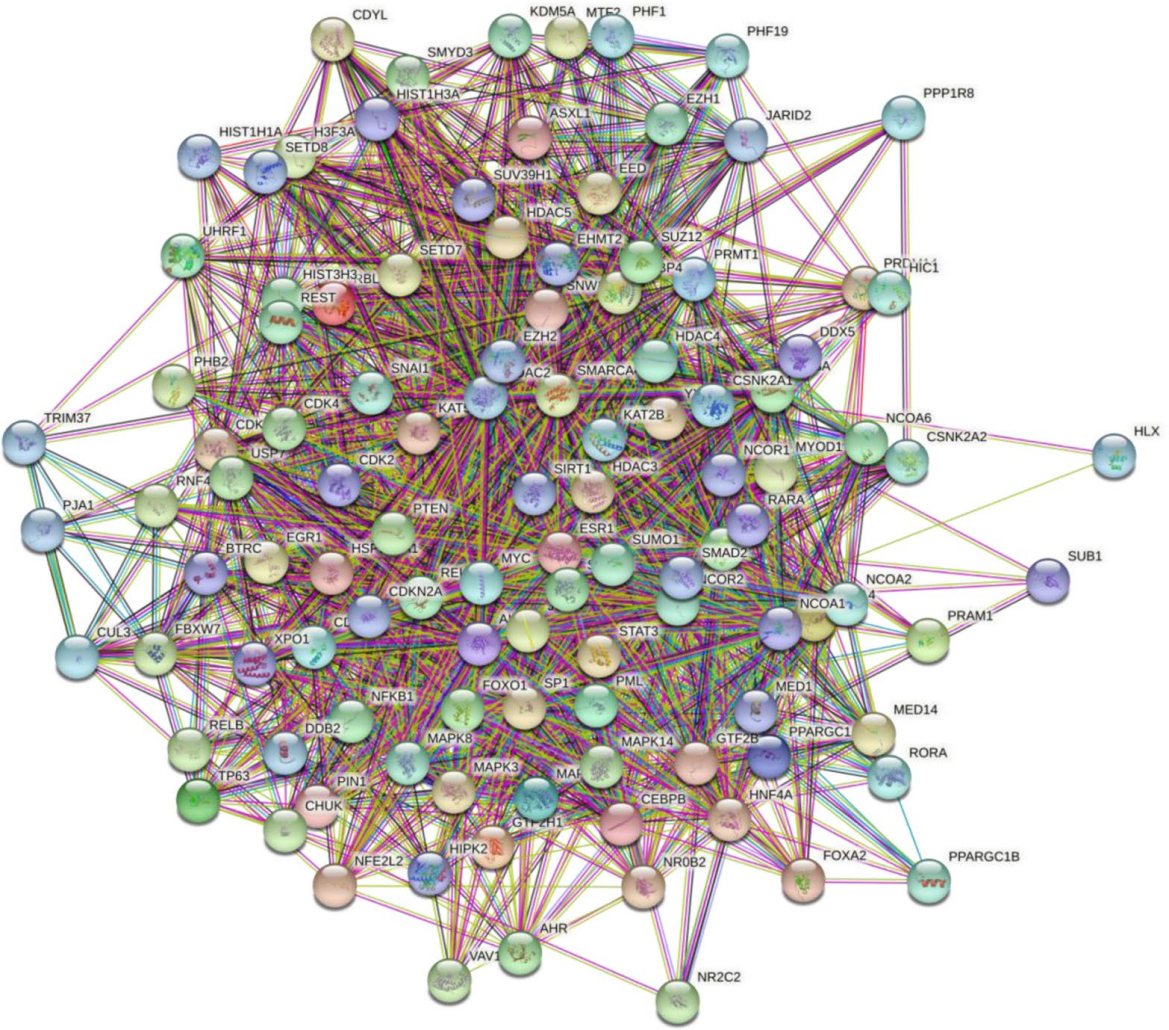
Inferred network of transcription factors, protein kinases and intermediate proteins connecting them for differentially expressed genes.

eXpression2Kinases (X2K)^26^ constructed a network of 110 proteins (Fig. 3) for the inputted list of differentially expressed genes. This network comprised the likely upstream TFs and PKs regulating DE genes.

Top 10 transcription factors in the network are presented in Table 3.

**Table 3.** Top 10 transcription factors in the inferred network.

Rank	Transcription factor	Hypergeometric *p* value	Differential gene expression	
			LogFC	*P* value
1	SUZ12, Polycomb Repressive Complex 2 Subunit (*SUZ12*)	1.60E−18	–	–
2	Hepatocyte Nuclear Factor 4 Alpha (*HNF4A*)	4.83E−18	− 12.881	2.63E−53
3	Myogenic Differentiation 1 (*MYOD1*)	2.75E−06	2.96357	4.95E−28
4	Estrogen Receptor 1 (*ESR1*)	0.001774	− 0.8383	0.04628
5	Nuclear factor, Erythroid 2 Like 2 (*NFE2L2*)	0.003565	− 0.9791	7.16E−09
6	Early Growth Response 1 (*EGR1*)	0.004111	–	–
7	Forkhead Box A2 (*FOXA2*)	0.0064	− 10.367	1.35E−31
8	RE1 Silencing Transcription Factor (*REST*)	0.00755	− 0.8824	0.00045
9	Tumor Protein p63 (*TP63*)	0.01508	1.05154	0.00077
10	Enhancer of Zeste 2 Polycomb Repressive Complex 2 Subunit (*EZH2*)	0.01646	–	–

All these transcription factors were found to be involved in processes associated with adipogenesis, or glucose homeostasis and seven of them were also differentially expressed (*P < 0.05*). Polycomb Repressive Complex 2 (PRC2) is involved in epigenetic silencing of *PPARγ* and two enriched transcription factors *SUZ12* and *EZH2*, as part of PRC2 negatively regulate its transcriptional activation^27^. Similarly, Hepatocyte nuclear factor 4α (*HNF4α*) together with its target *Hes6* maintains low expression level of *PPARγ* in liver^28^ so its expression in peripheral fat reflect poor adipogenesis. *MyoD* up-regulates *PPARγ* expression and has been reported to promote adipocyte differentiation^29^. *ESR1* (Estrogen Receptor 1) has been reported to physically associates with *PPARγ* and functionally interferes with its signalling^30^. The roles of Nuclear factor erythroid 2-related factor 2 (*NRF2*) in the regulation of metabolism, inflammation, mitochondrial physiology, and immune responses has been recognised^31^ and its modulation is extensively being studied for treatment of diseases that are caused by oxidative stress^32^. NRF2-knockout mice were found to be partially protected from high fat diet-induced obesity and developed a less insulin-resistant phenotype^33^. Transcription factor *EGR1* has also been reported to act as a negative regulator of 3T3-L1 adipocyte differentiation^34^. *FOXA2* is a negative regulator of adipocyte differentiation. It enhances the insulin receptor (*IR*), insulin receptor substrate-2 (*IRS2*), glucose transporter-4 (*GLUT4*), *HK2*, and *UCP2* and *UCP-3* genes in preadipocytes and adipocytes^35^. Another transcription factor *REST* has been reported to negatively regulate the expression of several neuronal genes, such as glutamate receptor 7 and synuclein γ and expression of these genes in adipocytes has been reported which suggests the role of *REST* in adipocyte differentiation and function in both white as well as brown adipose tissues^36^. The role of *TP65* is adipose biology is known and *PPARγ* agonists, including thiazolidinediones (TZDs) have been reported to induce anti-proliferation, differentiation, and apoptosis in adipocytes through inducing expression of endogenous or exogenous *TP63*^37^.

Three lipodystrophy-associated genes, Histone deacetylase 3 (*HDAC3*), Nuclear receptor subfamily 0 group B member 2 (*NR0B2*), and small ubiquitin-like modifier 1 (*SUMO1*) were also present in this network. *HDAC3* induces lipodystrophy by inhibiting glyceroneogenesis in adipocytes through repressing cytosolic phosphoenolpyruvate carboxykinase (*PEPCK*)^38^. *NR0B2* is transcriptionally regulated by *PPARγ* and has been found to be associated with insulin resistance^39^. *SUMO1* has been found to repress transcriptional activity of *PPARγ* through its sumoylation and consequently impairing its pro-adipogenic function^40^.

**Figure 4:**
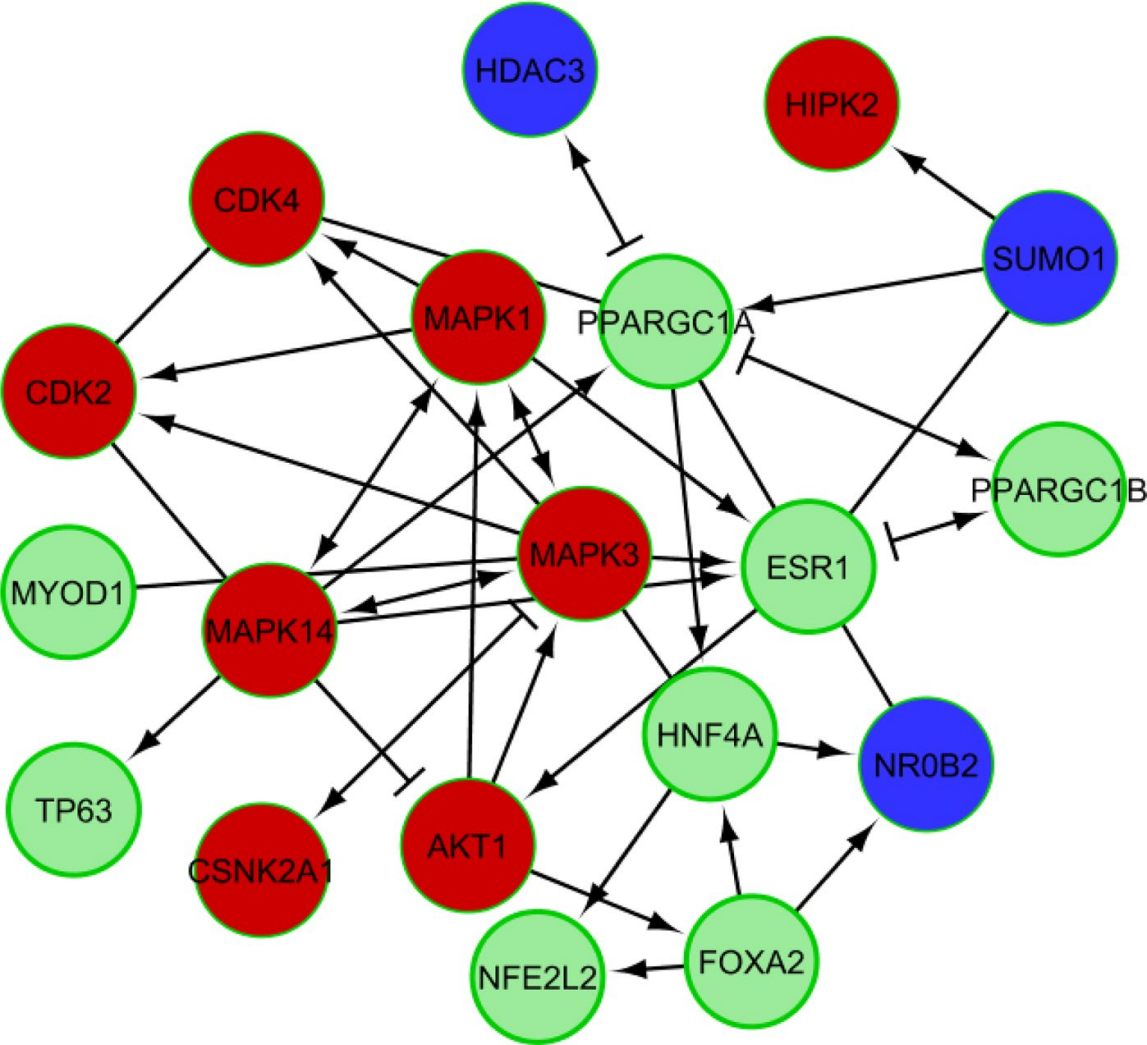
Derived minimal-molecular pathway based on the connectivity of top-ranked Transcription Factors (*Green*), Protein Kinases (*Red*), and Lipodystrophy genes (*Blue*).

Derived minimal network contain a total of 19 genes (Fig. 4) including eight kinases (*MAPK14, MAPK1, MAPK3, CSN2A1, HIPK2, AKT1, CDK2,* and *CDK4*), six transcription factors (*MYOD1, TP63, NFE2L2, FOXA2, HNF4A,* and *ESR1*), three lipodystrophy genes (*SUMO1, NR0B2,* and *HDAC3*), *PPARGC1A*, and *PPARGC1B.*

We further explored Type 2 Diabetes knowledge portal (https://t2d.hugeamp.org/) for these genes and found the association of *FOXA2, HNF4A, NR0L2, SUMO1, CDK2, MAPK3, HDAC3,* and *MYOD1* with glycemic, lipid, and anthropometric traits based on meta-analysis of multiple GWAS studies (Table 4).

**Table 4.** Association of genes in minimal network with relevant traits in multiple GWAS studies.

S. no.	Trait	Phenotype	Gene with GWAS *P* value
1	Type 2 Diabetes	Glycemic	*HNF4A* (*P-val* 1.602e−25), *MAPK3 *(*P-val* 3.868e−10*)*
2	Type 1 Diabetes		*CDK2* (*P-val* 5.318e−14)
3	Type 2 Diabetes adjusted BMI		*HNF4A* (*P-val* 7.982e−17)
4	Fasting Glucose		*FOXA2 *(*P-val* 1.282e−9)
5	Fasting Glucose adjusted BMI		*FOXA2 *(*P-val* 1.373e−9)
6	Random Glucose		*FOXA2 *(*P-val* 4.611e−23)
7	LDL-Cholesterol	Lipids	*HNF4A *(*P-val* 7.693e−25), *NR0L2 *(*P-val* 5.812e−28), *SUMO1 *(*P-val* 8.046e−20)
8	HDL-Cholesterol		*HNF4A *(*P-val* 1.35e−10)
9	Triglyceride		*HDAC3 *((*P-val* 3.204e−9),*NR0L2 *(*P-val* 1.159e−24)
10	Waist-hip ratio	Anthropometric	*HDAC3 *(*P-val* 4.501e−8), *MAPK3 *(*P-val* 1.581e−16), *MYOD1*(*P-val* 4.89e−8)

The gene *HNF4A* (Hepatocyte Nuclear Factor 4 Alpha) has been associated with monogenic autosomal dominant non-insulin-dependent diabetes mellitus type I and has been identified in GWAS studies in people of south Indian ancestrary alongwith another gene *TMEM163* (Transmembrane Protein 163)^41^. Its variants also showed association with decreased fasting plasma insulin and homeostatic model assessment of insulin resistance, indicating a plausible effect through impaired insulin secretion^42^.

A dual metric approach was then used to calculate an average score of node using degree-score, and bottleneck-score that reflects the importance of each node in this minimal network. Top 7 nodes are presented in Table 5. Three kinases: *MAPK3, MAPK14,* and *AKT1* were amongst these nodes.

**Table 5.** Ranking of nodes in minimal network based on their topological properties.

S. no.	Name	Score_degree	Score_bottleneck	Average score
1	*ESR1*	8	6	7
2	***MAPK3***	9	5	7
3	***MAPK14***	7	5	6
4	*PPARGC1A*	7	5	6
5	***AKT1***	5	2	3.5
6	*HNF4A*	5	2	3.5
7	*FOXA2*	4	1	2.5

All these kinases have been found to be associated with diabetes-related KEGG pathways—AGE-RAGE signaling pathway in diabetic complications (hsa04933), and Endocrine resistance (hsa01522). *MAPK3,* and *AKT1* were involved in Insulin signaling pathway (hsa04910), and Type II diabetes mellitus (hsa04930). *AKT1* is also involved in Adipocytokine signaling pathway (hsa04920), Regulation of lipolysis in adipocytes (hsa04923), Insulin resistance (hsa04931) pathways. Clearly their deregulation seems to play role in the T2D pathogenesis.

AKT Inhibitors are considered as very promising drug target in cancer research as dysregulation in phosphoinositide 3-kinase/AKT/mammalian target of rapamycin pathways have been found to associate with many cancers. However, due to connection of these pathways with insulin signalling, AKT Inhibitors have been reported to induce hyperglycaemia^43^ so their use as drug target in diabetic complications seems not appropriate. Inhibition of MAPK3 (ERK1) inhibitor in hypertrophic 3T3-L1 adipocytes has been reported to ameliorate the dysregulation of adipocytokine expression and suppressed the enhanced lipolysis activity. It has also shown to control blood glucose level in the diabetic mice^44^. A genome-wide association study suggests that MAPK14 is associated with diabetic foot ulcers^45^. MAPK14 (p38) is a stress-activated protein kinases (SAPK) and is involved in biological functions, such as inflammation, differentiation, proliferation, and apoptosis^46^. Pentosan polysulfate—an inhibitor of p38 MAPK pathway has been found to attenuate apoptosis and inflammation by down-regulating this pathway in high glucose treated human renal proximal tubular epithelial cells^47^.

## Discussion

In summary, this study find that in diabetics peripheral subcutaneous adipose tissue show molecular pathology characterized by differential expression of genes enriching glucose, lipid and protein metabolic pathways as well as adipogenesis, inflammation and coagulation related pathways. The imputed regulatory network of this transcriptome contained several transcription factors and protein kinases which converge on the process of adipogenesis. The majority of them were also differentially expressed in diabetics. Out of 52 differentially expressed miRNAs, eight are circulatory and are known to be associated with diabetes and its complications. Additionally few like miR-27a are also known to regulate the process of adipogenesis. Interestingly many of the regulatory factories were mapped to genetic loci previously showed association with diabetes and related intermediate phenotypic traits in genome wide association studies (GWAS) In other words these finding shed light on the regulatory mechanism and genome to phenome pathways of diabetes in peripheral subcutaneous adipose tissue.

Peripheral adipose tissue is generally considered protective in the pathogenesis of insulin resistance, but not so in the case of Asian Indians diabetics, who show thin fat phenotype. Recently we have reported that in diabetics peripheral subcutaneous adipose tissue on transcription profiling, show not only molecular pathology consistent with the so called “sick fat” or “adiposopathy”, but also its modules of co-expressed genes showed an association with several disease related intermediate phenotypic traits. In other words, these findings suggested the role of this adipose tissue pathology in the pathogenesis of insulin resistance and diabetes. Interestingly on functional analysis these modules of co-expressed genes enriched several adipogenesis and inflammation related pathways. These findings are consistent with the theory that it is the limitation of adipogenesis in subcutaneous adipose tissue and inability to expand and store excess fat lead to ectopic fat deposition in liver and muscle. The ectopic fat deposition in liver and muscle play an important role in the pathogenesis of insulin resistance and diabetes. The results of present study add to the current knowledge in this field by identification of network of transcription factors, protein kinases and miRNAs regulating this molecular pathology.

It was observed in this study that eight microRNAs known to be associated with T2D and its co-morbidities like diabetes retinopathy, and nephropathy were differentially expressed (*P* < 0.05) in diabetics.Over 214 DE genes, accounting to ~ 7% of all DE genes were being regulated by these microRNAs at stringent *P* < 0.01. Several of them in previous studies were found to be associated with insulin signalling, inflammation and adipogenesis, thereby suggesting their role, not only in sick fat or adiposopathy, but possibly also in the aetiology of cardiovascular and microvascular complications of diabetes. As most of them are circulatory miRNAs, they can not only serve as targets of new drug discovery, but also the biomarkers of adiposopathy. As a clinical indicator of adiposopathy such biomarkers can be better defined than the presently employed indicator—metabolic syndrome. Therefore, they deserve further investigations towards potential clinical diagnostic and therapeutic application.

Another important finding of this study is that a small network of 110 proteins, comprising upstream regulators of differentially expressed genes (i.e. transcription factors, protein kinases and intermediate proteins connecting them) was imputed on the basis of the differentially expressed genes. Interestingly more them 50% of them were also differentially expressed (*P* < 0.05). These results affirm the marked biological significance of this RNA-seq expression profiles. Interestingly, almost all of the top enriched upstream transcription factors are known to regulate genes implicated in the process of adipogenesis. Moreover, several of these genes had also shown linkage disequilibrium with diabetes related traits in previously published genome wide association studies. Therefore, these findings suggests that some of diabetes susceptibility genes possibly manifest clinically by influencing this regulatory network and control the process of adipogenesis in peripheral subcutaneous adipose tissue. Adipogenesis defects in peripheral subcutaneous adipose tissue can explain various aspects of “thin fat” phenotype like relatively thiner limbs (i.e. smaller mid arm circumference) in Asian Indian newborns, lesser fat mass in limbs of diabetics as compared to non-diabetics and relatively high insulin resistance for given body mass index in them etc^47^. Interestingly we recently observed that insulin resistance in diabetics showed positive correlation with adipocyte size (a parameter of poor adipogenesis) in the peripheral subcutaneous adipose depot, but not in the abdominal depots, thereby suggesting the important role of poor adipogenesis in the former adipose depot in the pathophysiology of diabetes in this population^49^. Identification of regulatory transcription factors, protein kinases and miRNAs in this study opens up the avenue for identification of thin fat phenotype specific biomarkers and molecular targets for drug development. In this regulatory network three lipodystrophy-associated genes were also present. As lipodystrophy is a monogenic disorder with Mendelian mode of transmission, whereas T2D is a complex multifactorial disease and its clinical picture is created by the interaction of several environmental and genetic factors such as frequent polymorphisms of multiple genes, still both exhibit similar pathophysiological alteration such as heightened insulin resistance, loss of peripheral fat, ectopic fat deposition, and adipocytokinemia etc. Presence of these genes therefore points toward the fact that thin fat phenotype might be a functional variant of lipodystrophy.

There are several potential clinical implications of the findings of this study. The higher diabetes risk among Asian Indians is generally attributed to central obesity, whereas this study find pathologic changes, particularly defects in adipogenesis in femoral adipose tissue in diabetics. Therefore, thigh related anthropometric parameter deserve further investigations as surrogate markers of adipose tissue pathology underlying diabetes mellitus in population. Moreover, targeting adipogenesis defects as a therapeutic strategy for the management of diabetes in population with drugs like pioglitazone is supported by the results of this study. Delineation of peripheral adipose tissue genome to phenome mechanism and pathways of insulin resistance support the concept of “adiposopathy” or “sick fat” as a formal disease for Asian Indians thin fat phenotype. The adiposopathy model of disease in addition to current gluco-centric diagnostic system has potential advantage of providing diagnostic frame for primordial prevention of diabetes.

There are several limitations of this study like relatively smaller sample size and only transcriptomic profile was generated. In order to precisely enlist potential biomarkers and novel drug targets, there is need of multi-omic investigations deciphering adipose tissue specific genome to phenome pathways and mechanisms of diabetes and insulin resistance in individuals showing thin fat phenotype. However, findings of this study, which to the best of our knowledge is the first report on regulatory mechanisms of peripheral adipose tissue dysfunction among Asian Indians, shed light on the pathways of genome to phenome correlation in diabetics.

In conclusions, this RNS-Seq study finds that peripheral subcutaneous adipose tissue among Asian Indians show pathology characterized by altered lipid, glucose and protein metabolism, adipogenesis defect and inflammation. A network of regulatory transcription factors, protein kinases and microRNAs have been imputed which converge on the process of adipogenesis. As the majority of these genes also showed altered expression in diabetics and some of them are also circulatory, therefore they deserve further investigation for potential clinical diagnostic and therapeutic applications.

## Methods

The study was conducted at Sawai Man Singh Medical College, Jaipur, India. Gluteofemoral fat biopsies were obtained from five normal glucose tolerants (NGT) and five T2D individuals. The study was approved by the ethical committee of SMS Medical College, Jaipur, India, and funded by the Indian Council of Medical Research (ICMR), New Delhi. Written informed consent was obtained from the participants prior to study. All methods were performed in accordance with the relevant guidelines and regulations. The inclusion and exclusion criteria were defined in Table 6.

**Table 6.** Inclusion and exclusion criteria for the study subjects.

Condition	Inclusion criteria	Exclusion criteria
Diabetic subjects	Non-obese (BMI < 30) type-2 diabetics diagnosed as per American Diabetes Association (ADA, 2012) criteria undergoing femur bone surgery	Presence of infection, malignancy and drugs affecting body fat/insulin resistance or adipokine expression like glitazones, metformin and glucocorticoids
Non-diabetic	Age and sex-matched non-obese (BMI < 30) normal glucose tolerance subjects undergoing femur bone surgery	Presence of infection, malignancy and drugs affecting body fat/insulin resistance or adipocytokines expression like glitazones, metformin and glucocorticoids. History of diabetes in first degree relatives

Total RNA was isolated from tissue biopsies using Qiagen RNeasy mini kit using manufacturer’s protocol. This procedure also captured few miRNA, tRNAs, rRNAs, lncRNAs in addition to mRNAs. QC of the extracted total RNA samples was carried out on Agilent Bioanalyzer and nanodrop.

mRNA paired end libraries were generated from each of the RNA samples using Illumina TruSeq mRNA library preparation kit. Sequencing was carried out on Illumina HiSeq 2500 using paired end chemistry of 2x100 bp read length. Total RNA samples with RIN value >7 were used for paired end library preparation using the TruSeq™ RNA Sample Preparation Kit version 3.0 (Illumina, Inc.) according to the manufacturer's instructions. Briefly, the Poly-A containing mRNA was purified from 2 μg of total RNA using oligo (dT) magnetic beads and was fragmented into 200–500 bp pieces in presence of divalent cations at 94°C for 5 min using an ultrasonicator. The cleaved RNA fragments were copied into first-strand cDNA using SuperScript-II reverse transcriptase (Life Technologies, Inc.) and random primers. After second- strand cDNA synthesis, fragments were end-repaired, A-tailed and indexed adapters were ligated. The products were purified and enriched with PCR to create the final cDNA library. The tagged cDNA libraries were pooled in equal ratios and used for 2Å ~ 100 bp paired-ends sequencing on a single lane of the Illumina HiSeq2500. After sequencing, the samples were de-multiplexed and indexed-adapter sequences were trimmed and other low quality bases were filtered or trimmed using in-house Perl scripts. Thus filtered, high quality reads (20x) were used for further analysis. The QC of the raw data was done using FastQC tool (http://www.bioinformatics.babraham.ac.uk/projects/fastqc/). A total of 4–8.5 GB data was generated for each of the samples.

The reads were then mapped to human reference sequence genome GRCh38.84 build using HISAT2^50^ with default parameters. Mapped reads were assembled into transcripts and quantified using StringTie^51^ and the annotated gtf file of GRCh38.84 build. Further all the mapped & quantified results of each samples were merged and abundances of each individual genes and transcripts were calculated using StringTie. Differential expression was calculated using DESeq2^52^ algorithms.

To check the reliability of RNA-seq analyses, qPCR (Real Time PCR) was done on ten genes—*AKT2, BCL6, LIPE, METTL9, CD59, LMNA, FOS, CCL2, MUSTN1,* and *ZNF638* in thigh (femoral) subcutaneous adipose tissue of four diabetic and four non-diabetic controls subjects in triplicates. The genes were randomly selected based on their important role in diabetes, insulin resistance, lipodystrophy, lipid storage and metabolism and in inflammatory pathways. The details are attached in supplementary sheet (S2). The oligonucleotide primer sequences used were raised using freely available online tools and synthesized as per appropriate melting temperature (Tm), GC%. A total of 2 μg of RNA was isolated from the thigh (femoral) subcutaneous adipose tissue biopsy using Qiagen RNeasy Mini Kit (Cat No. 74104) and quantified using the microfluidic-based capillary electrophoresis system (Bio-Rad Experion). cDNA Synthesis was done using QuantiNova Reverse Transcription Kit (Cat No. 205411) and real time PCR was done using Quanti Nova SYBR Green PCR Kit (Cat. No 208052).

For identification of enriched KEGG pathways by differentially expressed genes, STRING database (version 11.0) was used. To identify upstream transcription factors those regulating these differentially expressed genes, we used eXpression2Kinases (X2K) which derived an inferred network of transcription factors, protein kinases and intermediate proteins connecting them. A minimal-molecular pathway was derived from this network on the basis of connectivity of top-ranked TFs, PKs, PPARγ, and lipodystrophy genes and analyzed for therapeutic cues using network-based centrality measures like degree, and bottleneck using Cytohubba^53^.

For identification of differentially expressed miRNAs associated with diabetes, and related co-morbidities such as retinopathy, nephropathy, obesity, and inflammation, we searched Human MicroRNA Disease Database (HMDD v3.0)^54^ which manually collects a significant number of miRNA–disease association entries from literature. Identified miRNAs were then searched for their differentially expressed mRNA targets using Cytoscape^55^ through its app—CyTragetLinker.

## Supplementary Information

Supplementary Information 1.

Supplementary Information 2.

## References

[1] Khan MA, Hashim MJ, King JK, Govender RD, Mustafa H, Al Kaabi J (2020). Epidemiology of type 2 diabetes-global burden of disease and forecasted trends. J. Epidemiol. Glob. Health.

[2] Sniderman AD, Bhopal R, Prabhakaran D (2007). Why might South Asians be so susceptible to central obesity and its atherogenic consequences? The adipose tissue overflow hypothesis. Int. J. Epidemiol..

[3] Abate N, Chandalia M, Snell PG, Grundy SM (2004). Adipose tissue metabolites and insulin resistance in non-diabetic Asian Indian men. J. Clin. Endocrinol. Metab..

[4] Saxena A, Tiwari P, Wahi N, Soni A, Bansiwal RC, Kumar A (2019). Transcriptome profiling reveals association of peripheral adipose tissue pathology with type-2 diabetes in Asian Indians. Adipocyte.

[5] Porter SA, Massaro J, Homann U, Vasan RS, O’Donnel CJ, Fox CS (2009). Abdominal subcutaneous adipose tissue: a protective fat depot?. Diabetes Care.

[6] Bays H, Abate N, Chandalia M (2005). Adiposopathy: sick fat causes high blood sugar, high blood pressure and dyslipidemia. Future Cardiol..

[7] Saxena A, Tiwari P, Wahi N, Kumar A, Mathur SK (2020). The common pathophysiologic threads between Asian Indian diabetic’s “thin fat phenotype” and partial lipodystrophy: the peripheral adipose tissue transcriptomic evidences. Adipocyte.

[8] Maestro MA, Cardalda C, Boj SF, Luco RF, Servitja JM, Ferrer J, Scharfmann R, Shield JPH (2007). Distinct roles of HNF1 Β, HNF1 α, and HNF4 α in regulating pancreas development, Β-cell function and growth. Development of the Pancreas and Neonatal Diabetes.

[9] Cohen P (2002). Protein kinases—the major drug targets of the twenty-first century?. Nat. Rev. Drug Discov..

[10] Love MI, Huber W, Anders S (2014). Moderated estimation of fold change and dispersion for RNA-seq data with DESeq2. Genome Biol..

[11] Kanehisa M, Goto S (2000). KEGG: kyoto encyclopedia of genes and genomes. Nucleic Acids Res..

[12] Szklarczyk D, Gable AL, Lyon D, Junge A, Wyder S, Huerta-Cepas J, Simonovic M, Doncheva NT, Morris JH, Bork P, Jensen LJ (2019). STRING v11: protein–protein association networks with increased coverage, supporting functional discovery in genome-wide experimental datasets. Nucleic Acids Res..

[13] Kutmon M, Ehrhart F, Willighagen EL, Evelo CT, Coort SL (2018). CyTargetLinker app update: a flexible solution for network extension in Cytoscape. F1000Research.

[14] Chakraborty C, Doss CGP, Bandyopadhyay S, Agoramoorthy G (2014). Influence of miRNA in insulin signaling pathway and insulin resistance: micro-molecules with a major role in type-2 diabetes. Wiley Interdiscip. Rev..

[15] Assmann TS, Recamonde-Mendoza M, Souza BMD, Crispim D (2017). MicroRNA expression profiles and type 1 diabetes mellitus: systematic review and bioinformatic analysis. Endocr. Connect..

[16] Kaur K, Vig S, Srivastava R, Mishra A, Singh VP, Srivastava AK (2015). Elevated hepatic miR-22-3p expression impairs gluconeogenesis by silencing the Wnt-responsive transcription factor Tcf7. Diabetes.

[17] Gholaminejad A, Tehrani HA, Fesharaki MG (2018). Identification of candidate microRNA biomarkers in diabetic nephropathy: a meta-analysis of profiling studies. J. Nephrol..

[18] Herrera BM, Lockstone HE, Taylor JM, Ria M, Barrett A, Collins S (2010). Global microRNA expression profiles in insulin target tissues in a spontaneous rat model of type 2 diabetes. Diabetologia.

[19] Karolina DS (2012). Circulating miRNA profiles in patients with metabolic syndrome. J. Clin. Endocrinol. Metab..

[20] Lin Q, Gao Z, Alarcon RM, Ye J, Yun Z (2009). A role of miR-27 in the regulation of adipogenesis. FEBS J..

[21] Lenin R, Sankaramoorthy A, Mohan V, Balasubramanyam M (2015). Altered immunometabolism at the interface of increased endoplasmic reticulum (ER) stress in patients with type 2 diabetes. J. Leukoc. Biol..

[22] Jennewein C, von Knethen A, Schmid T, Brüne B (2010). MicroRNA-27b contributes to lipopolysaccharide-mediated peroxisome proliferator-activated receptor γ (PPARγ) mRNA destabilization. J. Biol. Chem..

[23] Zhu H, Leung SW (2015). Identification of microRNA biomarkers in type 2 diabetes: a meta-analysis of controlled profiling studies. Diabetologia.

[24] Caporali A, Meloni M, Völlenkle C, Bonci D, Sala-Newby GB, Addis R, Spinetti G, Losa S, Masson R, Baker AH, Agami R (2011). Deregulation of microRNA-503 contributes to diabetes mellitus–induced impairment of endothelial function and reparative angiogenesis after limb ischemia. Circulation.

[25] Pescador N, Pérez-Barba M, Ibarra JM, Corbatón A, Martínez-Larrad MT, Serrano-Ríos M (2013). Serum circulating microRNA profiling for identification of potential type 2 diabetes and obesity biomarkers. PLoS ONE.

[26] Clarke DJ, Kuleshov MV, Schilder BM, Torre D, Duffy ME, Keenan AB, Lachmann A, Feldmann AS, Gundersen GW, Silverstein MC, Wang Z (2018). eXpression2Kinases (X2K) Web: linking expression signatures to upstream cell signaling networks. Nucleic Acids Res..

[27] Sabatino L, Fucci A, Pancione M, Colantuoni V (2012). PPARG epigenetic deregulation and its role in colorectal tumorigenesis. PPAR Res..

[28] Martinez-Jimenez CP, Kyrmizi I, Cardot P, Gonzalez FJ, Talianidis I (2010). Hepatocyte nuclear factor 4α coordinates a transcription factor network regulating hepatic fatty acid metabolism. Mol. Cell. Biol..

[29] Deng B, Zhang F, Chen K, Wen J, Huang H, Liu W, Ye S, Wang L, Yang Y, Gong P, Jiang S (2016). MyoD promotes porcine PPARγ gene expression through an E-box and a MyoD-binding site in the PPARγ promoter region. Cell Tissue Res..

[30] Bonofiglio D, Gabriele S, Aquila S, Catalano S, Gentile M, Middea E, Giordano F, Andò S (2005). Estrogen receptor α binds to peroxisome proliferator–activated receptor response element and negatively interferes with peroxisome proliferator–activated receptor γ signaling in breast cancer cells. Clin. Cancer Res..

[31] He F, Ru X, Wen T (2020). NRF2, a transcription factor for stress response and beyond. Int. J. Mol. Sci..

[32] Dodson M, De La Vega MR, Cholanians AB, Schmidlin CJ, Chapman E, Zhang DD (2019). Modulating NRF2 in disease: timing is everything. Ann. Rev. Pharmacol. Toxicol..

[33] Chartoumpekis DV, Ziros PG, Psyrogiannis AI, Papavassiliou AG, Kyriazopoulou VE, Sykiotis GP, Habeos IG (2011). Nrf2 represses FGF21 during long-term high-fat diet–induced obesity in mice. Diabetes.

[34] Boyle KB, Hadaschik D, Virtue S, Cawthorn WP, Ridley SH, O'Rahilly S, Siddle K (2009). The transcription factors Egr1 and Egr2 have opposing influences on adipocyte differentiation. Cell Death Differ..

[35] Wolfrum C, Shih DQ, Kuwajima S, Norris AW, Kahn CR, Stoffel M (2003). Role of Foxa-2 in adipocyte metabolism and differentiation. J. Clin. Investig..

[36] Fuwa M (2019). 2003-P: the role of Re1-silencing transcription factor (REST) in adipocytes. Diabetes.

[37] Kim S, Lee JJ, Heo DS (2011). PPARγ ligands induce growth inhibition and apoptosis through p63 and p73 in human ovarian cancer cells. Biochem. Biophys. Res. Commun..

[38] Zhang J, Henagan TM, Gao Z, Ye J (2011). Inhibition of glyceroneogenesis by histone deacetylase 3 contributes to lipodystrophy in mice with adipose tissue inflammation. Endocrinology.

[39] Zhang Y, Hagedorn CH, Wang L (2011). Role of nuclear receptor SHP in metabolism and cancer. Biochim. Biophys. Acta Mol. Basis Dis..

[40] Simon DN, Domaradzki T, Hofmann WA, Wilson KL (2013). Lamin A tail modification by SUMO1 is disrupted by familial partial lipodystrophy–causing mutations. Mol. Biol. Cell.

[41] Tabassum R, Chauhan G, Dwivedi OP, Mahajan A, Jaiswal A, Kaur I, Bandesh K, Singh T, Mathai BJ, Pandey Y, Chidambaram M (2013). Genome-wide association study for type 2 diabetes in Indians identifies a new susceptibility locus at 2q21. Diabetes.

[42] Kooner JS, Saleheen D, Sim X, Sehmi J, Zhang W, Frossard P, Been LF, Chia KS, Dimas AS, Hassanali N, Jafar T (2011). Genome-wide association study in individuals of South Asian ancestry identifies six new type 2 diabetes susceptibility loci. Nat. Genet..

[43] Crouthamel MC, Kahana JA, Korenchuk S, Zhang SY, Sundaresan G, Eberwein DJ, Brown KK, Kumar R (2009). Mechanism and management of AKT inhibitor-induced hyperglycemia. Clin. Cancer Res..

[44] Ozaki KI, Awazu M, Tamiya M, Iwasaki Y, Harada A, Kugisaki S, Tanimura S, Kohno M (2016). Targeting the ERK signaling pathway as a potential treatment for insulin resistance and type 2 diabetes. Am. J. Physiol. Endocrinol. Metab..

[45] Meng W, Veluchamy A, Hébert HL, Campbell A, Colhoun HM, Palmer CN (2017). A genome-wide association study suggests that MAPK 14 is associated with diabetic foot ulcers. Br. J. Dermatol..

[46] Barros, J. B., da Silva Santos, R., da Silva Reis, A. A. Implication of the MAPK signalling pathway in the pathogenesis of diabetic nephropathy. *Diabetes* (2019).

[47] Chen P, Yuan Y, Zhang T, Xu B, Gao Q, Guan T (2018). Pentosan polysulfate ameliorates apoptosis and inflammation by suppressing activation of the p38 MAPK pathway in high glucose treated HK 2 cells. Int. J. Mol. Med..

[48] Misra A (2015). Body fat patterning, hepatic fat and pancreatic volume of non-obese Asian Indians with type 2 diabetes in North India: a case-control study. PLoS ONE.

[49] Yajnik C, Fall C, Coyaji K, Hirve SS, Rao S, Barker DJ (2003). Neonatal anthropometry: the thin-fat Indian baby. The Pune Maternal Nutrition Study. Ind. J. Obes. Relat. Metab. Disord..

[50] Kim D, Langmead B, Salzberg SL (2015). HISAT: a fast spliced aligner with low memory requirements. Nat. Methods.

[51] Pertea M, Pertea GM, Antonescu CM, Chang TC, Mendell JT, Salzberg SL (2015). StringTie enables improved reconstruction of a transcriptome from RNA-seq reads. Nat. Biotechnol..

[52] Love MI, Huber W, Anders S (2014). Moderated estimation of fold change and dispersion for RNA-seq data with DESeq2. Genome Biol..

[53] Chin CH, Chen SH, Wu HH, Ho CW, Ko MT, Lin CY (2014). cytoHubba: identifying hub objects and sub-networks from complex interactome. BMC Syst. Biol..

[54] Huang Z, Shi J, Gao Y, Cui C, Zhang S, Li J, Zhou Y, Cui Q (2019). HMDD v3.0: a database for experimentally supported human microRNA–disease associations. Nucleic Acids Res..

[55] Shannon P, Markiel A, Ozier O, Baliga NS, Wang JT, Ramage D, Amin N, Schwikowski B, Ideker T (2003). Cytoscape: a software environment for integrated models of biomolecular interaction networks. Genome Res..

